# All-Light Remote Driving and Programming of Soft Actuator Based on Selective Laser Stimulation and Modification

**DOI:** 10.3390/polym17101302

**Published:** 2025-05-09

**Authors:** Jingjing Zhang, Hai Hu, Wenliang Liang, Zhijuan Fuyang, Chenchu Zhang, Deng Pan

**Affiliations:** 1 Anhui University Center for Applied Mathematics, School of Mathematical Sciences, Anhui University, Hefei 230601, China; jjmath@ahu.edu.cn; 2 Information Materials and Intelligent Sensing Laboratory of Anhui Province, Anhui University, Hefei 230601, China; wd24301028@stu.ahu.edu.cn (H.H.); b24301105@stu.ahu.edu.cn (W.L.); b23201045@stu.ahu.edu.cn (Z.F.); 3 Anhui Province Key Lab of Aerospace Structural Parts Forming Technology and Equipment, Institute of Industry and Equipment Technology, Hefei University of Technology, Hefei 230009, China

**Keywords:** all-light remote driving, laser patterning, reprogrammable actuator, bionic soft robot

## Abstract

Soft robots are advantageous due to their flexibility, ability to interact with humans, and multifunctional adaptability. However, developing soft robots that are unrestrained and can be reprogrammed for reversible control without causing damage remains a significant challenge. The majority of soft robots have a bilayer structure with internal stress, which limits their motion to pre-programmed anisotropic structures. Taking inspiration from pillworms found in nature, we propose an approach for controlling and reprogramming the motion of actuators using infrared light as the driver and a laser-melted paraffin wax (PW) shell as the controller. The dual-purpose shell can not only protect the actuator but can also alter its initial motion behavior to achieve multiple programming, profile modeling, object grasping, and directional crawling tasks, thereby enabling active changes to the motion strategy in response to external stimuli. This method can also be extended to other materials with similar properties and multi-stimulus responses, offering a new pathway for developing unconstrained, autonomous soft robots and intelligent devices.

## 1. Introduction

Soft actuators have garnered significant attention due to their unique advantages, including flexibility, adaptability, and ability to convert various external stimuli—such as electricity [1,2,3], light, heat [4,5], magnetic fields [6], pH [7], chemicals [8], air pressure [9,10,11], and humidity [12,13]—into mechanical deformation outputs. These capabilities enable soft actuators to perform diverse tasks such as crawling [14,15,16], rolling [17], jumping [18,19], gripping [20], camouflage [21], and information encryption [22]. Consequently, they have been widely applied in fields such as bionic soft robots [23,24,25], intelligent wearable electronics [26,27], and artificial muscles [28,29].

Despite these advantages, a major challenge in soft actuator development lies in achieving reversible and precise control over their deformation shapes. Reversibility and precision are crucial for practical applications [30], and most soft actuators rely on smart materials with shape memory properties [31]. One common approach to control deformation is to incorporate anisotropic microstructures, such as those produced by UV lithography, printing, or painting [32,33,34,35,36]. These structures enable programmable deformation by altering structural orientation. However, these methods are often complicated, expensive, and lack repeatability. Additionally, the inability to remotely reprogram actuators during operation significantly limits their versatility.

**Figure 1:**
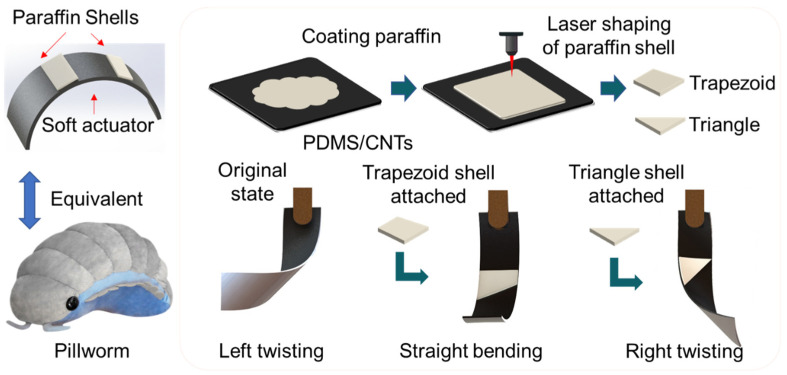
The schematic diagram of the pillworm-inspired soft actuator and the process of programming PW shells. The original state of the actuator is left-twisting. After attaching the trapezoid shell and triangle shell, the bending states of the actuator change to straight-bending and right-twisting.

The proposed approach for driving and reprogramming soft actuators is inspired by the natural behavior of pillworms (Figure 1), which respond to external stimuli and protect themselves with a shell. This method involves applying paraffin wax (PW) to the PDMS/CNT bilayers to create a semi-rigid shell that can be melted and reshaped using a laser. The PW exhibits excellent thermoplasticity, allowing it to melt under laser heating and form specific shapes. These shapes adhere to the bilayer structure, creating rigid supports. By altering the shape of the PW shell, the support angle can be controlled, thereby changing the initial bending direction of the bilayer soft actuator. The photothermal action of the laser not only induces actuator movement but also facilitates the reprogramming of the PW shell to achieve different deformation patterns.

Compared to conventional actuators, actuators with PW shells offer several advantages. Compared to conventional actuators, an actuator with a PW shell offers reprogrammability and enhanced structural control. The PW not only serves as a semi-rigid shell to protect the active layer but also enables programmable motion via localized laser melting and reshaping, thereby unlocking new actuation modes (e.g., off-axis bending, direction switching) and real-time trajectory adjustment. Thanks to its excellent plasticity, the PW shell can be melted and solidified repeatedly, enabling reversible deformation and reprogramming. Furthermore, the wireless nature of light-driven programming allows remote driving, programming, and reprogramming of soft robots.

## 2. Design and Qualitative Analysis of Programmable Soft Actuator

### 2.1. The Design Process and Driving Mechanism for Actuators

**Figure 2:**
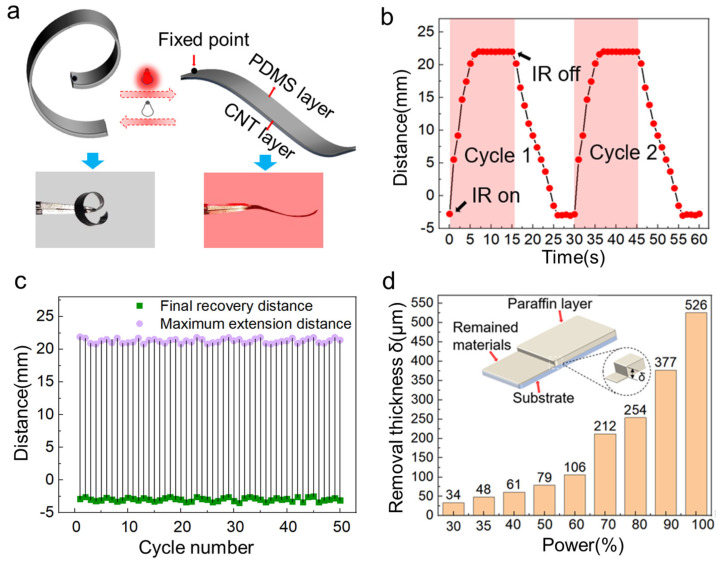
Process of the actuator design and driving mechanism. (**a**) Schematic and physical diagram of the actuator deformation before and during illumination. (**b**) Diagram of the actuator extension and contraction distance difference under two stimulus cycles. (**c**) Fatigue test of the actuator under 50 illumination cycles. (**d**) Relationship of laser power and the removed thickness of PW shell.

PDMS/CNTs‘ bilayer material is a high-performance active material with rapid photothermal response. Benefitting from the good photothermal conversion efficiency of the CNTs and the high thermal expansion coefficient of the PDMS, the bilayer material is capable of large deformation when subjected to heat. As shown in Figure 2a, the bilayer structure can stretch, which is similar to the curling response of the pillworm when stimulated. This is due to the release of thermal stresses, which are formed during the manufacturing of the material, as mentioned in Figure S1. When irradiated by near-infrared (NIR) light, the CNT layer absorbs heat and generates more stress than the PDMS layer to make the bilayer gradually bends toward the CNT side. The actuation mechanism of our system is based on a light-to-heat-to-deformation conversion pathway. Specifically, the embedded CNTs function as efficient photo-thermal transducers in visible and near-infrared spectrums, converting incident light into localized heat. According to Figure 2b, the material stretched to its maximum extent within 6 s when irradiated by infrared light, then returned to its original form in 12 s when the light was turned off. As shown in Figure 2b, when the IR light is turned off, the actuator gradually returns to its original shape. The observed plateau in the response curve between 25 and 30 s and 55 and 60 s corresponds to the actuator having fully recovered and maintaining a stable configuration. After 50 cyclic irradiations, as shown in Figure 2c and Figure S2, the bilayer film exhibited steady reversible bending performance without fatigue.

Paraffin wax was chosen as the shell material for the bilayered actuator. The main constituents of microcrystalline PW are branching saturated hydrocarbons with high viscosity and ductility. PW with a melting point of 80° was deposited on a glass slide, as shown in Figure S3. When the substrate was placed on a heat plate set at 100 °C, the PW rapidly melted to liquid condition. Two drops (100 µL) of black pigment were added to the melted PW and thoroughly stirred to increase the light absorption of the PW layer during laser processing. The CNT/PDMS bilayer material was spread flat onto a slide and cut into a rectanglar shape by a laser direct writing system. Then, the melted black PW was drop-casted on the CNT layer of the actuator. At room temperature, the PW solidified instantly on the surface of CNT layer to form a PW shell with a thickness of ~52 µm. This thickness was obtained by drop-casting 100 μL of molten paraffin and was measured using a laser profilometer (KEYENCE VK-X250, Keyence (Japan) Co., Ltd., Osaka, Japan). The chosen thickness balances mechanical support with reprogrammability via laser melting. As the PW shell covered the whole surface of the actuator, the actuator was kept straight and did not bend under the light stimulus. As mentioned in Figures S4 and S5, the nanosecond laser is able to remove excess paraffin and produce a variety of complicated forms on PW substrates, such as letters, bar codes, patterns, etc. In order to find the ideal laser processing parameters of removing the excess PW, a 600 µm thick layer of PW was attached to a flat CNT substrate. Then, the PW layer was removed by different laser powers to record the removed thicknesses *δ* (Figure 2d). The full energy output of the nanosecond laser was 60 W. Through the experiment, the influence of nanosecond laser power on the removed thickness *δ* was established. The optimal laser power value was 21.6 W to remove the PW shell with a thickness of 52 µm.

### 2.2. Effect of Different Widths of PW Layers on the Bending Performance of the Actuators

**Figure 3:**
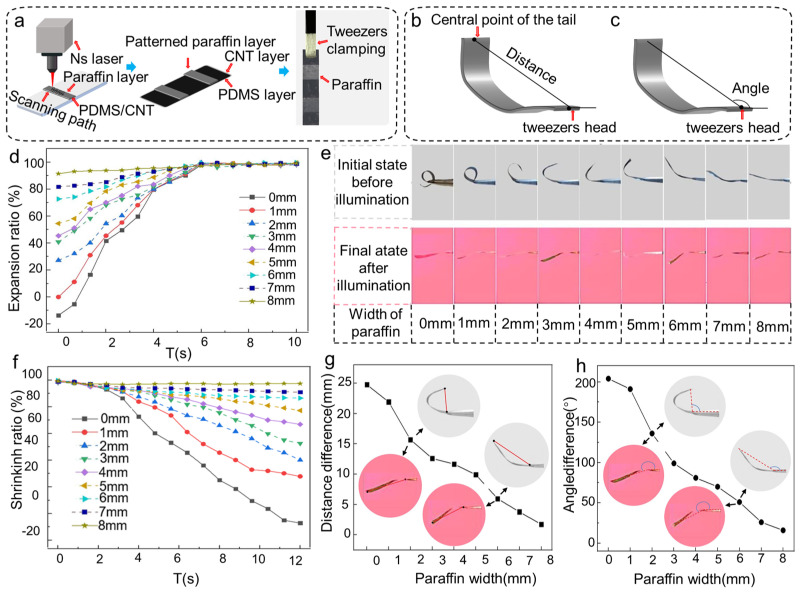
Effect of various widths of PW on the bending performance of actuators. (**a**) Process of the nanosecond laser removal of the excess PW. (**b**) Denotation of the actuator extension distance and bending angle under infrared light illumination. (**c**) The bending angle (α) is defined as the angle between the line connecting the actuator’s free end and the tweezer tip and the longitudinal axis of the tweezers. This angle serves as the primary parameter for quantifying actuator deformation. (**d**) Expansion ratio of each group of actuators under illumination with respect to time. (**e**) Comparison of the bending state of each group of actuators before and after illumination. (**f**) Contraction ratio of each group of actuators after ceasing illumination in relation to time. (**g**) Deviation in the extension distance of each group of actuators before and after illumination. (**h**) Difference in the bending angle of each group of actuators before and after illumination.

The shape of PW shells can change the twisting state of the actuators. To quantitatively investigate the influence of the PW shells on the photothermal response of the actuator, shells with various widths were applied to the bilayers. The actuators with sizes of 24 mm × 4 mm were covered by PW shells with various widths. The shape of shells were controlled by nanosecond laser scanning, as shown in Figure 3a. After laser processing, a pair of PW shells, which were symmetrical and parallel to each other, were formed on the actuator at the head and tail. The widths of PW shells ranged from 1 mm to 8 mm, and an actuator without PW shells was used as a control sample, denoted as 0 mm. The actuators were held by tweezers at 2 mm from the top and placed horizontally, with the PDMS side facing up. As shown in Figure 3b, the extension distance *L* and bending angle *α* were used to calculate the degree of bending. *L* is the distance from the center point of the head to the center point of the tail, and *α* represents the angle formed by the extension direction and the horizontal line. The photothermal response of the actuator was evaluated using an NIR light set directly above the actuator at 15 mm. The expansion ratio of the actuator and the contraction ratio of the actuator are expressed as follows [37]:

(1) $$\varepsilon =(L/L_{0})\times 100\%,$$

where the value of *L*_0_ is 22 mm, which is the distance from the midpoint of the tip of the tweezers to the midpoint of the tail of the actuator. *L* refers to the current length of the actuator (measured from the clamping point to the tail).

As shown in Figure 3c, to better comprehend the deformation process of the structured soft actuator made from the bilayer material, the bending angle (α) is defined as the angle between the line connecting the actuator’s free end and the tip of the tweezers (clamping point) and the longitudinal axis of the tweezers.

As depicted in Figure 3d, the expansion ratio of the actuator increased with the width of the attached PW shells, and the time to stretch to the maximum expansion ratio was ~5.8 s, with the maximum expansion ratio being around 97%. The maximum extension angles were all about 190°, as illustrated in Figure 3e, with tiny variances owing to the gravity of the PW shells. As presented in Figure 3f, the contraction ratio and the ability of the actuator to recover to the initial state decreased with the increase in the width of the PW shells. This phenomenon is primarily caused by the weight of the PW gradually surpassing the recovery stress of the material when the width of the PW shells exceeds 2 mm. The difference in extension distance and bending angle between the initial and final state was inversely related to the width of the PW, as shown in Figure 3g,h. The above results show that the gravity and covering area of the PW are critical parts of the photothermal reaction of the actuator. Additionally, the shapes of PW shells can also influence the deformation behavior of soft actuators.

### 2.3. Influence of Different Tilt Angle PW Structures on Actuator Direction Change

**Figure 4:**
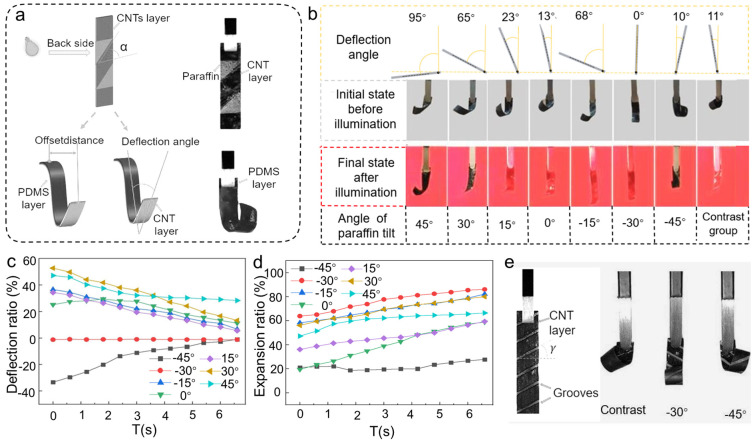
Effect of different PW-shell tilt angles on the actuator deflection. (**a**) Representation of deflection distance and the deflection angle of the actuator after attachment of PW. (**b**) Comparison of deflection status of each actuator group before and after infrared light illumination. (**c**) Deflection ratio of each actuator group under illumination with regard to time. (**d**) Expansion ratio of each actuator group under illumination in relation to time. (**e**) Diagram of the deflection state of the etched grooves on the surface of the material.

The PW shells can not only have impact on the expansion and shrinkage of actuators but can also influence the deformation directions when placed tilted to the edge of the actuators. As shown in Figure 4a, the PW shells were cut into parallelograms with inclination angles. Here, the angle of inclination was defined as the angle between the edge of the pattern and the horizontal direction (taken as positive for counterclockwise and negative for clockwise). Due to the asymmetry of the CNT layer during the manufacturing process, the actuator without the PW shells was generally deflected to the left at an angle of ~12° in its initial state. The PW shells were patterned on the CNTs‘ surface, with inclination angles varying from −45° to 45° with a 15° gradient. The deflection distance *d* and the deflection angle *β* were used to calculate the deflection of the actuator; *d* is the length of the line connecting the center point of the tweezer’s gripping tip and the center point of the actuator end in the horizontal direction, and *β* is the angle between the midline and the perpendicular line. The midline is determined as the line from the center point of the head to the tail of the actuator. As shown in Figure 4b, the larger the PW‘s tilt angle, the larger the initial deflection angle. In addition to the visual comparisons in Figure 4b, a quantitative summary of the deflection angles under different PW inclination angles is provided in Figure S6 (Supporting Information), showing a consistent trend between the tilt pattern and off-axis bending behavior. As the PW shells tilted from left to right, the actuator also gradually deflected from left to right. In particular, the initial deflection angle of the actuator was ~0° when the tilt angle was −30°, indicating that the original deflection force of the actuator equaled the force generated by the tilted PW shell. When the tilt angle was greater than −30°, the actuator began to deflect to the right to achieve a reversal of direction. The deflection ratio of the actuator can be expressed as follows:

(2) $$\gamma =(d/L_{0})\times 100\%$$

Here *d* is the horizontal displacement between the center of the actuator tip and the clamping point, and *L*₀ is the original actuator length.

Under NIR illumination, the offset ratio of each group of the actuator gradually converged toward the zero line, as illustrated in Figure 4c. The offset ratio eventually stabilized after 6 s of irradiation. The duration for attaining the maximum expansion extent was practically the same, as shown in Figure 4d, but the direction of infrared illumination coincided with the initial deflection of the actuators, resulting in different maximum extension degrees for each actuator. The direction of deflection of the actuator can be well-manipulated by regulating the tilt angles of the PW shells. Additionally, processing anisotropic microgrooves can also change the deflection angle of the actuator, as shown in Figure 4e. A portion of the CNT layer was removed by laser, resulting in the formation of grooves on the CNT layer. The inclination angle of the grooves were 30° and 45°, respectively. The results show that the microgrooves on the CNT layer also have the effect of altering the deflection direction. This form of processing, however, disrupts the structure of the bilayer material itself, making it impossible to reprogram it. To further evaluate the mechanical output performance of the actuators, the deformation force measurements were conducted on samples with a fixed width of 2 mm and varying lengths of 16 mm, 18 mm, 20 mm, and 22 mm. The comparison between the actuators with and without PW shells is shown in Figure S7. The results indicate that the deformation force increases with actuator length across the tested range, which is consistent with the expected mechanical amplification due to longer active segments. Notably, the presence of the PW shell slightly reduces the output force, which is attributed to its additional mass and damping effect. However, the overall trend of increasing force with length remains unchanged, confirming that the PW shell does not compromise the actuator’s intrinsic actuation capability. These findings support the actuator’s applicability in practical scenarios where mechanical interaction with the environment is required.

### 2.4. Reprogrammable Design of the Actuator

**Figure 5:**
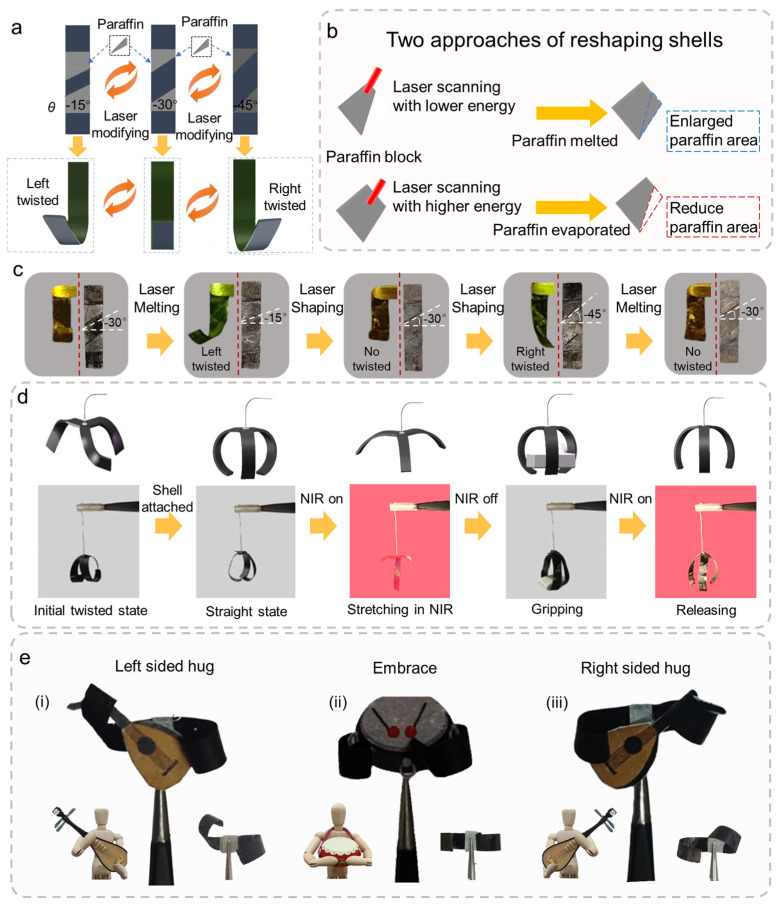
Reversible programming design of the actuator. (**a**) Schematic diagram of the actuator‘s reversible programming. The twisting states can be switched by laser-modifying the shape of paraffin shells. (**b**) The laser-modifying strategy of enlarging and reducing the area of shells. Shells can be enlarged by melting paraffin and can be reduced by evaporating paraffin. (**c**) A single circle of the reversible programming of the soft actuator. (**d**) Actuator with shells can act as a gripper to grasp and release objects under the NIR. (**e**) Actuators with different twisting states can realize left hug, embrace, and right hug by changing the attached shells.

Based on the experiment results above, a reprogramming approach of soft actuators was proposed in Figure 5a. Using an actuator with tilted PW shells with an angle of −30°, the actuator bent straight in the initial state and the PW shells could be reshaped by laser to increase or decrease the deflection angles of the actuators. The actuator twisted to the right when the tilt angle was −45° and twisted to the left when the angle was −15°. The tilt angle of PW can be altered between these three angles, resulting in a change in the actuator‘s twisting direction. The tilt angle of the PW was altered by a nanosecond laser-based method (Figure 5b). To decrease the tilt angle of the PW shell, a laser with low power was utilized to perform rapid cycle scanning the PW shell. Then, the entire PW was quickly melted. Subsequently, one end of the processing platform was elevated to make the liquid PW flow under the effect of gravity. When the paraffin flowed to the designated position, the laser stopped scanning to make the paraffin solidify into the desired shape rapidly. When increasing the tilt angle of the PW, a laser with higher power was applied to remove the excess directly. By converting between the two processing methods, the shape of the PW could be alternated several times, thus realizing multi-directional deflection of the actuator. The reprogramming process involves the repeated localized melting and resolidification of the paraffin wax (PW) shell under NIR laser exposure. During this process, a small amount of PW may gradually evaporate or redistribute, which may lead to slight degradation of structural fidelity over time. In our experiments, up to 4–5 bending direction changes could be reliably achieved before noticeable loss of function occurred. This demonstrates that the actuator is capable of multiple reprogramming cycles, although the reusability is ultimately limited by the stability of the PW material under repeated photothermal cycling.

A whole reversible programming cycle was shown in Figure 5c. The twisting states of the actuators were changed from straight to left twist, back to straight, then to right twist, and finally back to straight, while the tilt angles of the PW shells varied from −15° to −45°. The experimental results demonstrated that the actuator can be programmed to repeatedly switch the twisting states without compromising the performance characteristics.

Complex deformation can be achieved by combining several actuators with predefined PW shells. Figure 5d and Movie S1 show a clamp with three jaws, where the actuator was cut into a three-jaw shape and linked to the handle. The initial state of the clamp was like an elongated grass with the three jaws twisted, which made the clamp unable to grip objects due to a lack of mechanical strength. After adding PW shells with −30° tilt angles, the clamp had a claw-like shape. Due to the attachment of the PW, the jaws were more robust and capable of withstanding greater weight, which is three times the mass of the actuator. When the NIR was switched on, the jaws opened to release the object. By splicing two rectangular actuators with different PW shells together, complex light-driven deformations can be formed (Figure 5e). The actuator was converted into a vertical bend after PW shells with −30° tilt angles were applied. After reducing the tilt angle to −45°, the actuator bent in the opposite direction and unfolded in a straight line under infrared light. These three stretching states are like a player holding a lute or a drum, forming a three-piece orchestra.

## 3. Application of Programmable Soft Actuators

**Figure 6:**
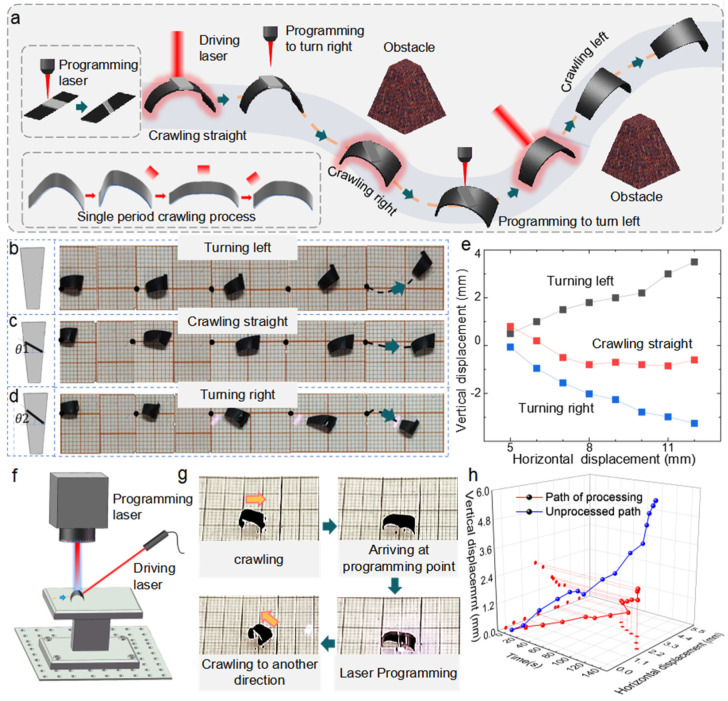
Application of programmable soft actuators. (**a**) Schematic diagram of the crawling actuator with full optical control to avoid obstacle movement and a brief description of the crawling mechanism of the actuator. (**b**) Diagram of the original crawling path of the actuator. (**c**) Diagram of the actuator crawling along a straight line. (**d**) Diagram of the actuator crawling along a right line. (**e**) The trajectories of the actuators are left turn (actuator in (**b**)), straight (actuator in (**c**)), and right turn (actuator in (**d**)). (**f**) The process of the all-light reprogramming and driving of the actuator. (**g**) The actuator first crawls to the programming point, then is programed by laser-scanning the paraffin shell to form another twisting state and crawls in another direction. (**h**) Comparison of the trajectory of the original and programmed actuators.

Based on the above research, we propose a crawling actuator motion scheme, as shown in Figure 6a, in which only optical control is used throughout the motion process without other contact forms. When the actuator crawls in a certain trajectory to the programming point, the shape of the PW shells are modified by laser to make the actuator crawl in another direction to avoid the obstacle. An NIR laser with a wavelength of 808 nm was applied as the driving laser. The focus of the laser was a 3 × 1.5 mm^2^ square, with the output power ranging from 0 to 300 mW. The output power was set to 250 mW to guarantee no thermal damage to the actuator and optimal driving efficiency.

The actuator initially bent downwards, and as the laser scanned along the actuator’s midline, the actuator moved to the side with more friction due to the distinction in friction between the head and tail of the actuator. Thus, the crawling actuator moved forward and turned to one side due to the asymmetric bending. The original motion path of the actuator without PW shells is shown in Figure 6b and Movie S2, with horizontal and vertical displacements of 13.5 mm and 5 mm, respectively, over 160 s. The actuator was designed in the shape of an isosceles trapezoid to provide greater asymmetric friction to enhance its maneuverability. The lengths of the top and lower bases were 2 mm and 4 mm, respectively, with a distance of 10 mm between the two bases. Using the qualitative analysis in Section 2.2 and Section 2.3, we propose a solution that enables the asymmetric bending actuator to move in variable directions. It was found that setting the width of the attached PW shell to 0.5 mm did not impair the crawling speed of the actuator. PW shells with tilt angles of −30° and −45° were selected to compensate for the initial actuator’s deflection angle and to accomplish considerable steering. The trajectory of the actuator with a PW with a −30° tilt angle is shown in Figure 6c and Figure S8 and Movie S3. The actuator crawled along a straight line for 13 mm in 160 s with relatively minimal vertical displacement. As shown in Figure S8, the actuator without a PW shell exhibited a forward-left crawling trajectory, while the addition of a PW shell with a 30° inclination realigned the actuator’s motion to a straight-forward direction. This demonstrates the programmable steering capability enabled by the PW structure. By adding PW shells with a tilt angle of −45°, the direction of the actuator shifted to right (Figure 6d and Movie S4). As the tilt angle increased, the initial degree of actuator retraction increased, and the distance between the head and tail shrank, resulting in an increase in the motion step per irradiation period. In this way, the crawling speed of the actuator with PW shells of −45° was significantly increased, achieving a horizontal displacement of 9 mm and a vertical displacement of 4 mm in 56 s. The crawling trajectory is shown in Figure 6e with the right-side end of the actuator set as the observation point. It is obvious that the crawling path can be effectively controlled by the shape of the PW shell. Figure 6f shows the driving and reprogramming procedure of the actuator as follows: the actuator with PW shells with a tilt angle of −30° crawled in a straight path driven by the NIR laser. When crawling to the programming location, the NIR laser rapidly scanned from the head to the tail of the actuator to flatten it. Then, the nanosecond laser was activated to reshape the PW to form an actuator with a new crawling direction. In the end, the actuator crawled to the new direction via the NIR laser. The whole process of the driving and programming can be divided into four parts: the beginning state, crawling to the processing point, nanosecond laser programming, and the new crawling direction, as shown in Figure 6g. The difference in crawling trajectory between the actuator with and without the PW shell is shown in Figure 6h and Movies S5–S7; the PW attached to the actuator is removed as black smoke. The center point of the actuator was used as a reference point. The experimental results reveal that by adding anisotropic PW shells, the direction and speed of the actuator can be precisely regulated. In all the experiments, the driving laser was manually controlled. Although the precision of hand-held laser operation was sufficient in this work to achieve reliable actuation and directional programming, more precise systems—such as galvanometric scanning mirrors—can be employed in future studies to enable more complex and automated motion control.

## 4. Conclusions

The approach for driving and programming soft actuators by adhering shell structures to a photothermal actuator using all wireless lasers is low-cost, convenient, and non-destructive. The crawling speed and offset direction of soft actuators can be altered by varying the paraffin’s width and tilt angle. In contrast with conventional actuators, it can be programmed more effectively by employing completely optical contactless operation, which has the capacity to easily adjust predicted trajectories for varied terrains. More than that, the programming can be implemented during the execution of tasks and is harmless to the actuator. The proposed actuator can be integrated into soft robotic platforms as programmable micromanipulators, optical-controlled valves in microfluidics, or bio-inspired grippers in confined environments. Its all-optical programmability and wireless operation make it particularly suitable for closed-loop light-controlled systems.

## 5. Methods

Preparation of materials: The PDMS (polydimethylsiloxane) solution (Sylgard 184 silicone elastomer) was purchased commercially from Hangzhou Bald Advanced Materials Co., Ltd. (Hangzhou, China); the cured PDMS film was used for heat folding and bodily support. The ink (S630) used to form the carbon layer on the surface of the PDMS film was purchased commercially from Ningbo Deli Stationery Co., Ltd. (Ningbo, China). Paraffin pigments (black, liquid oil soluble colorants) were commercially obtained from XuZhou Da Hong Trading Co., Ltd. (Xuzhou, China) to boost paraffin heat absorption during laser processing, minimize brittleness, and improve adherence to the material.

Fabrication of the rolled PDMS/CNT bilayer: A magnetic agitator was used to agitate the liquid–poly PDMS matrix and the curing substance (ratio 10:1) for 5 min at 2000 rpm. To eliminate air bubbles, the homogeneous mixture was poured into a Petri dish and put in a vacuum room for an hour. The PDMS solution was evenly spin-coated onto the subatrates, then cured for 30 min at 100 °C before being rinsed and desiccated with anhydrous ethanol. Carbon ink was dripped onto the PDMS film’s surface, and graphite was distributed evenly to form a carbon layer on the PDMS surface. The carbon-coated PDMS sheet was baked at 60 °C for 30 min before being peeled off the slide to create a PDMS/CNT bilayer. The initial condition of the bilayer exhibited a curled structure due to the differential in stress between the two faces of the bilayer.

Nanosecond laser-focused patterning processing system: A frequency-tripled, Q-switched, single-mode, neodymium-doped yttrium aluminum garnet nanosecond laser (Spectra-Physics) with a wavelength of 1064 nm, a repeat frequency of 100 KHz, a scanning speed of 3500 mm/s, and a pulse diameter of 10 ns serves as the irradiation source for the focusing laser.

IR lamp and NIR laser: The various bending performance tests of the actuator were performed under vertical irradiation of infrared light with a Philips infrared lamp of 100 W in the wavelength range of 600–2800 nm, purchased from Ohaka Yu Runxiang Medical Co., Ltd. The motion of the looper-like crawling was driven by near-infrared light irradiation (808 nm, 0~300 mW, Anford, Shenzhen, China).

## Data Availability

The original contributions presented in this study are included in the article/Supplementary Material. Further inquiries can be directed to the corresponding author.

## Supplementary Materials

The following supporting information can be downloaded at: https://www.mdpi.com/article/10.3390/polym17101302/s1: Figure S1. PDMS/CNTs bilayer preparation process. Curing of PDMS layer and carbon layer requires placing in an oven and using a spatula to peel the material from the substrate. Figure S2. Side view of the stretching state of the double-layer material under infrared illumination corresponding to four different time periods and its infrared thermogram. Figure S3. Macroscopic and microscopic diagrams of the ideal shape obtained using nanosecond laser modification of paraffin wax. Figure S4. The nanosecond laser patterns various complex shapes on paraffin substrates with a thickness of approximately 52 μm, and the excess paraffin can be ideally removed. Among them, the width of the thinnest bar in the barcode is 0.4 mm. Figure S5. Microscopic view of nanosecond laser processing of differently spaced grooves in paraffin-attached material. Figure S6. Quantitative analysis of bending angles of actuators under different inclination angles of the paraffin wax (PW) shell. Figure S7. The deformation force of the actuator. Figure S8. Comparison of motion trajectory of actuator without paraffin and with paraffin. Movie S1. Three-claw actuator grips the object and releases it under infrared light. Movie S2. Trajectory of an actuator without paraffin attachment driven by NIR laser. Movie S3. Trajectory of an actuator with a 30° tilt angle paraffin driven by NIR laser. Movie S4. Trajectory of an actuator with a 45° tilt angle paraffin driven by NIR laser. Movie S5. Trajectory of the actuator without paraffin attachment before and after Ns laser processing under the machining platform. Movie S6. Main view of the trajectory of the paraffin-attached actuator before and after Ns laser processing under the machining platform. Movie S7. Auxiliary view of the trajectory of the paraffin-attached actuator before and after Ns laser processing under the machining platform.

## Abbreviations

The following abbreviations are used in this manuscript:

PW	paraffin wax
PDMS	polydimethylsiloxane
CNTs	carbon nanotubes
NIR	near infrared
