# Supplementary material for: All-Light Remote Driving and Programming of Soft Actuator Based on Selective Laser Stimulation and Modification
Source: Polymers (Basel). 2025 May 9;17(10):1302. doi: 10.3390/polym17101302 (PMC12115092; doi:10.3390/polym17101302)
Supplement: Supplementary file 1 [file polymers-17-01302-s001.zip › Supporting Information.pdf]

Supporting Information

All-light remote driving and programming of milli-meter scale soft actuator based on selective laser stimulation and modification

This file includes:

Supporting Figures 1 to 8

Supporting Movies 1 to 7

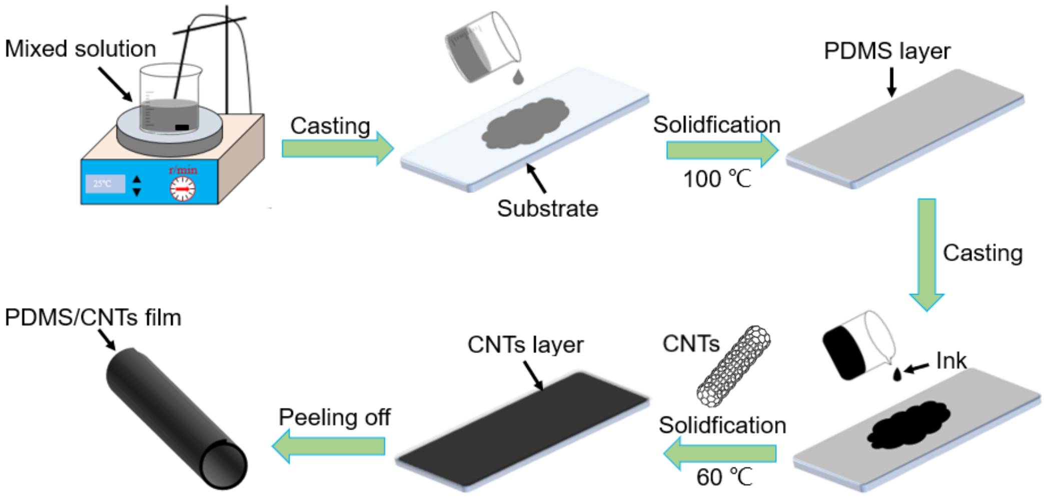

Figure S1. PDMS/CNTs bilayer preparation process. Curing of PDMS layer and carbon layer requires placing in an oven and using a spatula to peel the material from the substrate.

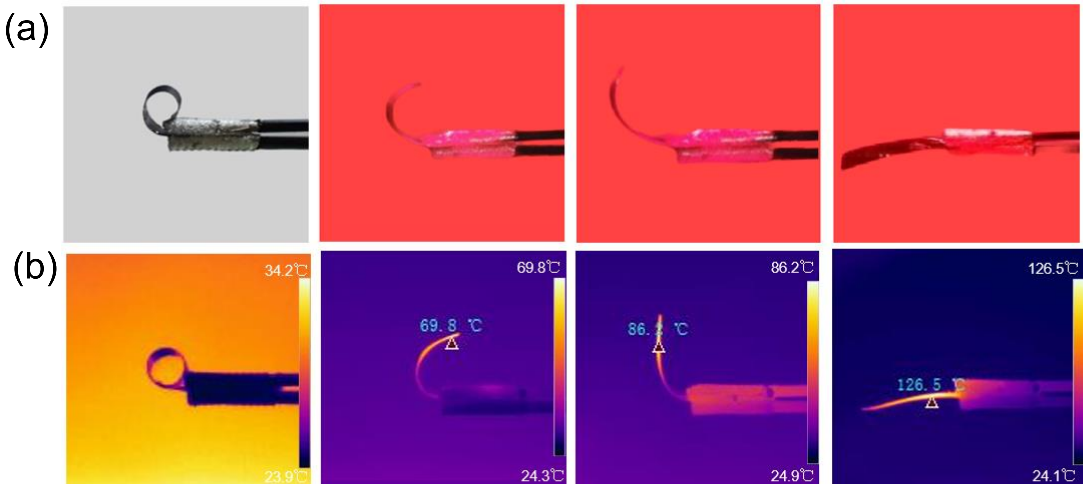

Figure S2. Side view of the stretching state of the double-layer material under infrared illumination corresponding to four different time periods and its infrared thermogram.

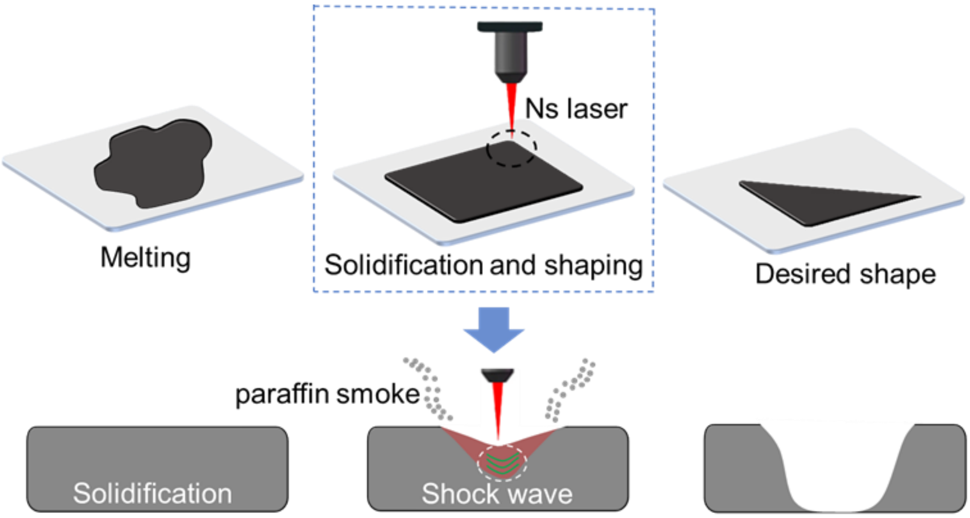

Figure S3. Macroscopic and microscopic diagrams of the ideal shape obtained using nanosecond laser modification of paraffin wax.

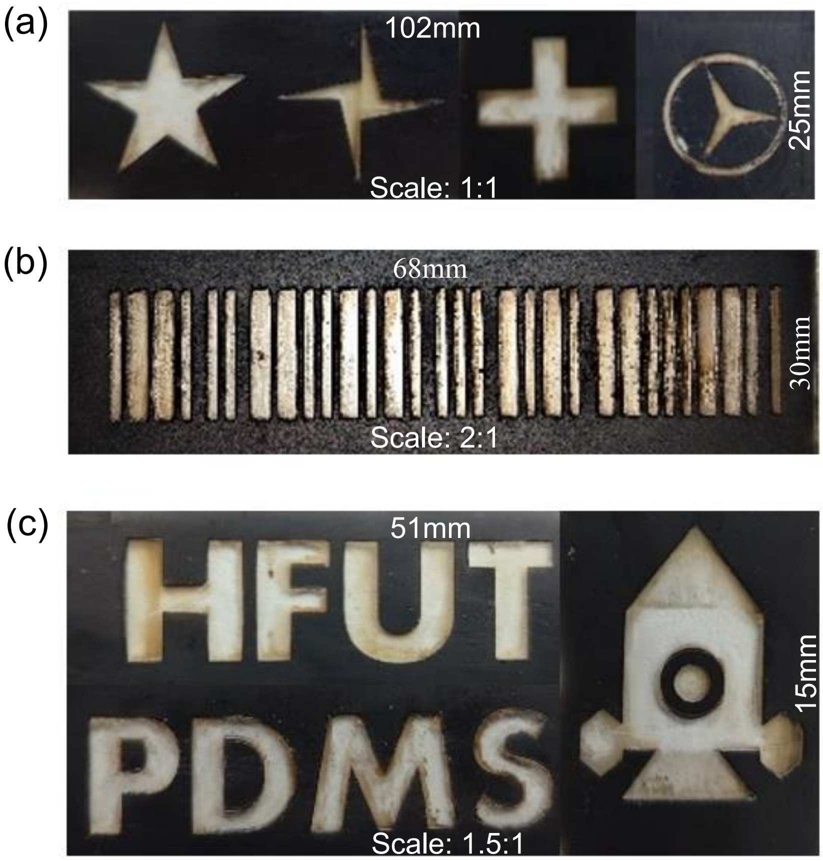

Figure S4. The nanosecond laser patterns various complex shapes on paraffin substrates with a thickness of approximately 52 μm, and the excess paraffin can be ideally removed. Among them, the width of the thinnest bar in the barcode is 0.4mm.

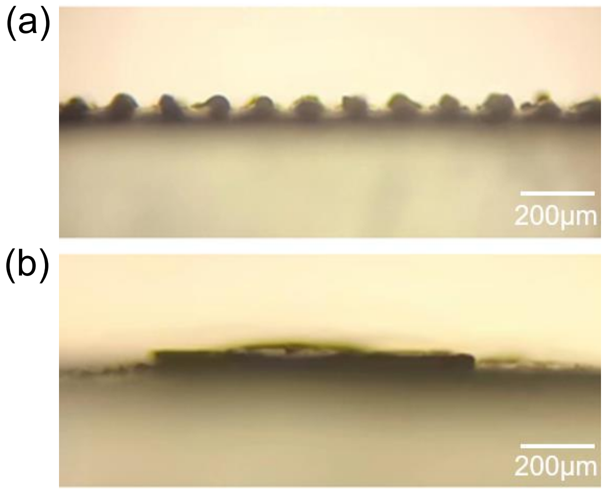

Figure S5. Microscopic view of nanosecond laser processing of differently spaced grooves in paraffin-attached material. a) Microscopic view at a laser scan pitch of 100 μm. b) Microscopic view at a laser scan pitch of 1μm, paraffin wax is well removed from the surface of the material and the material suffers very little thermal damage.

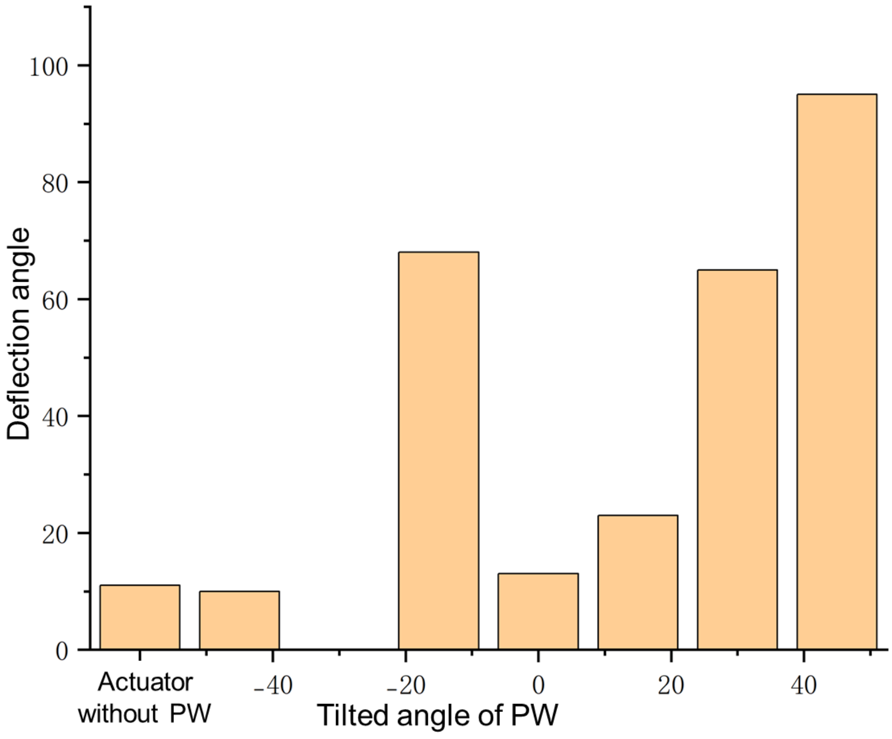

Figure S6. Quantitative analysis of bending angles of actuators under different inclination angles of the paraffin wax (PW) shell.

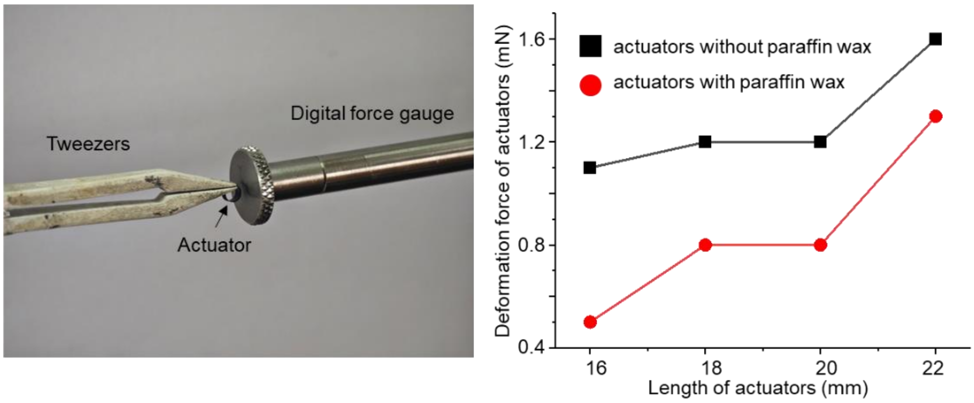

Figure S7. The deformation force of the actuator. (a) Photograph of the experimental setup used for deformation force measurement. The actuator is fixed at one end and pressed against a high-sensitivity microforce sensor under NIR illumination. The deformation force is recorded by a Mark-10 M5-012 digital force gauge. (b) Table of measured deformation forces for actuators with a width of 2 mm and varying lengths (16 mm, 18 mm, 20 mm, and 22 mm), both with and without paraffin wax (PW) shells. The data show that longer actuators produce higher forces, while the presence of the PW shell slightly reduces the output force without altering the overall trend.

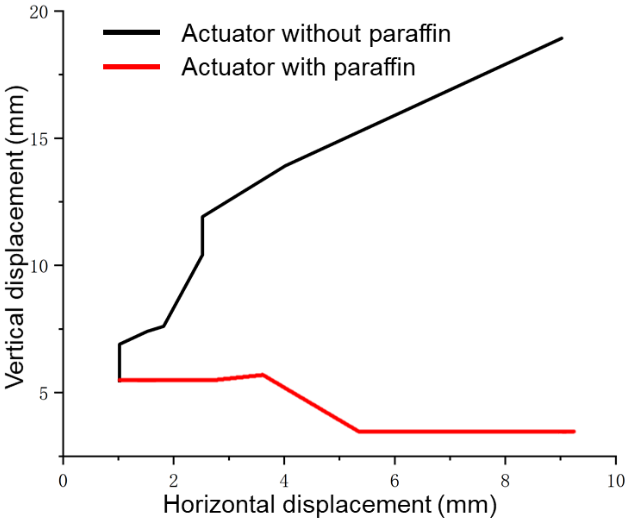

Figure S8. Comparison of motion trajectory of actuator without paraffin and with paraffin. a) Transverse and longitudinal coordinate variation of an actuator without paraffin attachment driven by NIR laser. b) Transverse and longitudinal coordinates of an actuator with an inclination angle of 30° paraffin driven by NIR laser.

Supporting Movies

Movie S1. Three-claw actuator grips the object and releases it under infrared light.

Movie S2. Trajectory of an actuator without paraffin attachment driven by NIR laser.

Movie S3. Trajectory of an actuator with a 30° tilt angle paraffin driven by NIR laser.

Movie S4. Trajectory of an actuator with a 45° tilt angle paraffin driven by NIR laser.

Movie S5. Trajectory of the actuator without paraffin attachment before and after Ns laser processing under the machining platform.

Movie S6. Main view of the trajectory of the paraffin-attached actuator before and after Ns laser processing under the machining platform.

Movie S7. Auxiliary view of the trajectory of the paraffin-attached actuator before and after Ns laser processing under the machining platform.
